# Climate change and disease in plant communities

**DOI:** 10.1371/journal.pbio.3000949

**Published:** 2020-11-24

**Authors:** Jeremy J. Burdon, Jiasui Zhan

**Affiliations:** 1 CSIRO Agriculture & Food, Canberra, Australian Capital Territory, Australia; 2 Department of Forest Mycology and Plant Pathology, Swedish University of Agricultural Sciences, Uppsala, Sweden; University of York, UNITED KINGDOM

## Abstract

Climate change is triggering similar effects on the incidence and severity of disease for crops in agriculture and wild plants in natural communities. The complexity of natural ecosystems, however, generates a complex array of interactions between wild plants and pathogens in marked contrast to those generated in the structural and species simplicity of most agricultural crops. Understanding the different impacts of climate change on agricultural and natural ecosystems requires accounting for the specific interactions between an individual pathogen and its host(s) and their subsequent effects on the interplay between the host and other species in the community. Ultimately, progress will require looking past short-term fluctuations to multiyear trends to understand the nature and extent of plant and pathogen evolutionary adaptation and determine the fate of plants under future climate change.

## Abstract 2

Climate change is triggering similar effects on the incidence and severity of disease for crops in agriculture and wild plants in natural communities. However, this Essay maintains that accounting for the complexity of wild systems is a vital part of fully understanding the potential impact of climate change, not only on individual pathogen species but, more importantly, on entire natural communities.

## Introduction

Climate change is the most challenging environmental issue facing humankind today. Many plant and animal communities are now experiencing rapid and significant changes in temperature, rainfall, evaporation patterns, and a dramatic increase in the occurrence of extreme events [1]. From an agricultural point of view, these changes have major implications for the geographic distribution of crops and their associated pathogens and the extent and reliability of production. For natural ecosystems and communities, the implications are perhaps less well understood but are arguably even more concerning, given the potential to precipitate a cascade of effects: changes in the fate of an individual pathogen species—an increase or a decrease leading to local extinction—may be surpassed by consequent increases or decreases in host fitness, generating “knock-on” effects in the structure of whole plant communities. Our ability to mitigate such changes is far more limited in natural systems than in agricultural systems. Humans have at least some control over the size and impact of pathogen populations in agriculture through breeding, agronomy, nutrient and moisture manipulation, and the use of chemical controls. Essentially, none of these interventions are practically feasible or environmentally acceptable in natural plant communities.

## Features of the changing patterns in agriculture and forestry

Much has been written about the likely impact of climate change on disease incidence and severity, epidemic frequency, and its spatial distribution in agriculture [2–4]. Such studies make a range of epidemiological assumptions based on observations of current disease triangles (host–pathogen–environment) and predictions of climate change. However, the disease triangle of any plant–pathogen interaction may shift in response to climate change, favoring different climatic preferences and niche breadths in the future [5]. Furthermore, while some environmental changes, such as increasing temperature and changing precipitation, are linked directly to changes in pathogen incidence and severity [6], others, such as increases in CO_2_ levels, have more indirect effects by changing biomass production, the density of crop stands, and hence humidity within the canopies [7].

Interest in the consequences of climate change on pathogen population dynamics in agricultural systems is understandable. History has demonstrated that massive crop losses can occur when climatic conditions are particularly conducive to disease development. We may well expect such outbreaks to occur more often in some production areas either due to invasion of new pathogens or due to increases in the severity of existing pathogens. Yet, although major epidemics may occur, humans will be able to ameliorate impacts in agricultural systems through manipulation of host genetic factors, environmental parameters, planting time, or pesticide applications. Less consideration has been given to the eco-evolutionary consequences of climate change for crops and pathogens. In areas where changes favor the pathogen, genetic change in invasive traits such as aggressiveness (and/or infectivity), fungicide sensitivity, and eco-niche breadth and preference [5,8,9] can occur rapidly. Equally, there is evidence supporting the possibility of genetically based temperature adaptation [10] that may make the prospect of expansion of a pathogen’s geographic range more concerning. However, human-directed evolution can ensure corresponding changes in the defensive traits of crops.

Studies of climate change and plant pathogens have placed considerable emphasis on agricultural cropping systems and, to a lesser extent, the future role of pathogens in semi-managed forestry production systems. In the forestry literature, there is a slow accumulation of examples in which diseases have been detected considerably closer to the pole, usually further north, than was previously the case [11]. Some of these outbreaks may simply reflect exotic invasion events triggered by accidental pan-national transportation through trade or tourism, particularly those involving diseases caused by short-dispersal pathogens such as bacteria and nematodes. In other cases, the appearance of pathogens outside their established geographic range or increased activity within a range [12] appears to reflect climate change modifying the adaptation of pathogens, for example, a major outbreak of *Diplodia sapinea* on *Pinus sylvestris* in Sweden [13]. Not surprisingly, and somewhat akin to agricultural systems, most of these forestry studies focus on the effect of changing climatic conditions on the population dynamics of the relevant pathogen and the health and survival of the host tree [14].

## Features of the changing patterns in natural ecosystems

Ecological assessments of climate change impacts have strongly focused on phenology and range distribution of plants, birds, insects, and other animals. Phenological shifts associated with first flowering, first appearance of migratory insects and birds, and breeding dates occur in many species but not in all [15,16]. Poleward and increasing altitudinal movement are expected to be a common future event as already observed in some plants and animals [17,18].

The potential for climate change to disrupt predator–prey and plant–insect interactions has been recognized [19], but potential impacts on pathogens in natural plant communities and any associated ecological consequences have attracted little interest except with respect to trees, where forestry studies have dominated. However, every plant species has at least 1 associated pathogen, and most are host to many more. Pathogens, as well as pests and other herbivores, greatly influence the fecundity and longevity of individual plants, the dynamics of whole plant populations, the evolution of species, and the structure and dynamics of whole communities.

The major role that pathogens play in shaping the nature and structure of individual species and whole communities has become clear over the past 50 years [20,21]. This impact may seem to be more limited to forest trees and other longer-lived species that remain present, and recordable, long after death than for herbaceous species that disappear quickly, leaving no evidence of their previous existence. Yet, careful studies provide a clear picture of pathogens affecting the size [22,23] and genetic structure of individual host populations [24] and that greater host diversity leads to reduced disease impact and pathogen evolution [25,26].

**Fig 1:**
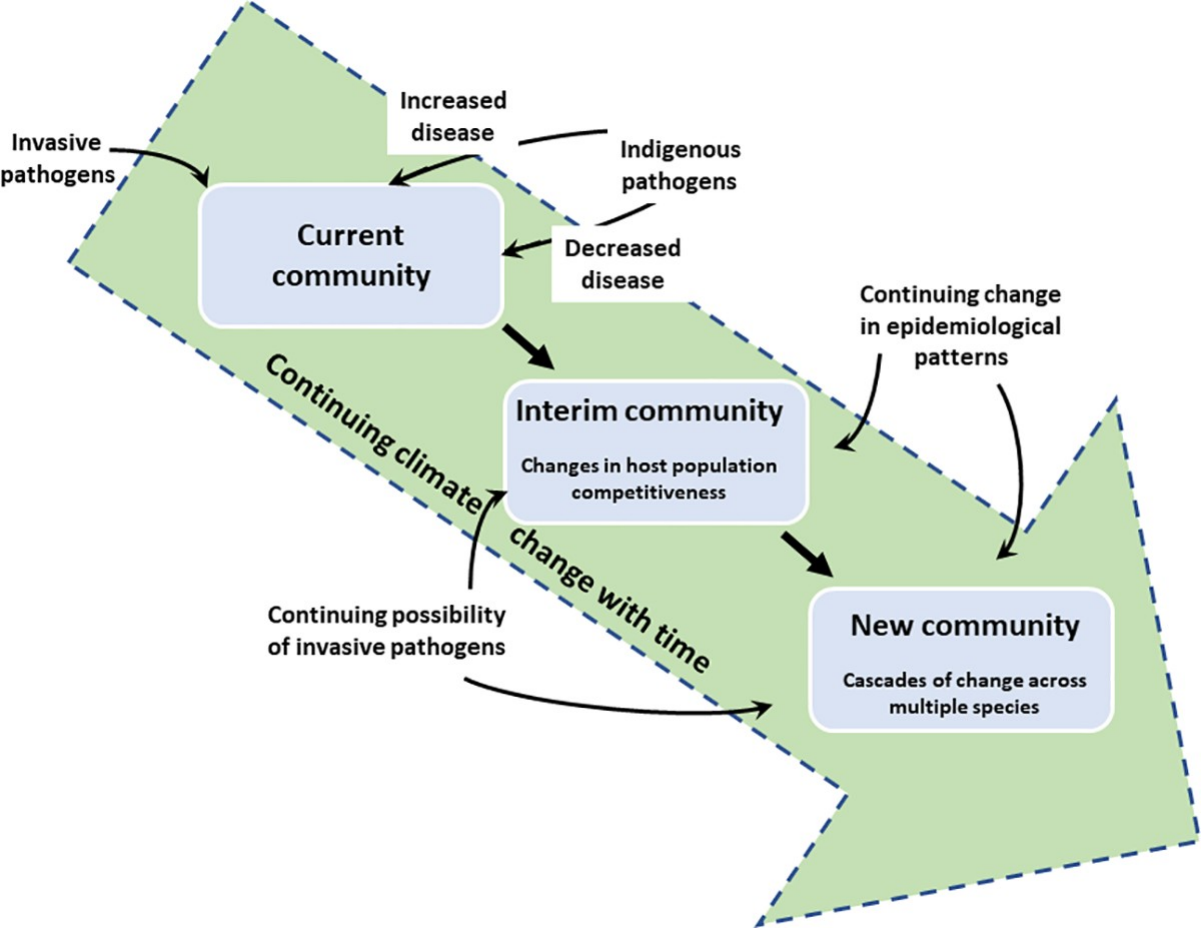
Cartoon showing ways in which climate change may affect wild plant community structure as increased or decreases in disease or invasion of novel pathogens leads to either positive or negative changes in host fitness. In turn, these changes may trigger a cascade of changes between host and nonhost species with consequential changes in community structure.

This is the situation today—what of the future? Environmental conditions affect disease severity in agricultural settings and wild systems alike. For this reason, we expect pathogens to behave similarly between agricultural and wild systems while recognizing that the longer-term community outcomes may be quite different. Most wild plants and their associated pathogens live in groups of loosely connected individual patches called metapopulations. The spatial structuring and potential variation in local habitat quality of these metapopulations—which differ markedly from agricultural systems—as well as the relative efficiency of both pathogen and host in responding to climate change can be important in determining local plant–pathogen dynamics [27,28]. Smaller, more fragmented plant populations show lower disease incidence and severity than do larger, more consolidated populations. As the consequences of climate change—particularly regarding temperature and precipitation—play out through time, we will see many unpredicted changes in both host plant and pathogen populations and in the communities they inhabit (Box 1, Fig 1).

**Box 1. Case studies involving population and community change driven by changing climate**

**Local pathogen extinction tied to rising temperature**

Climate change’s potential effects on disease epidemiology in a natural setting is exemplified by a 26-year-long study of a metapopulation of approximately 230 *Filipendula ulmaria* host patches in a northern Swedish archipelago. There, the host is infected by rust (*Triphragmium ulmariae*) that varies in incidence and severity from year to year and patch to patch. These long-term data demonstrate a strong correlation between an increased rate of local extinction of the pathogen and a steady rise in summer temperatures [29]. Furthermore, host populations without disease show a greater growth rate than those with disease. Thus, the local extinction of this pathogen could potentially have much wider impacts by changing community structure.

**Changing snow cover alters snow blight incidence and generates community change**

Increasing temperatures have led to a marked decline in Northern Hemisphere snow cover that has accelerated over the last 50 years [30,31]. Such changes have great significance for the long-term dynamics of snow blight fungi, revealing the potential for far-reaching community change. Snow blights are caused by a range of fungal pathogens that spread from plant to plant below the winter snowpack in many boreal and alpine plant communities. Careful examination of their impact shows that they may markedly affect the density and patterning of *Pinus sylvestris* stands [32], whereas their impact on young *Pinus cembra* populations appears to play a major role in determining the transition from meadow to conifer forest in the Alps [33]. More subtle effects are found in subarctic ecosystems where the density of ericaceous dwarf shrubs is affected by epidemics of snow blight fungi that cause considerable plant mortality [34]. In the case of the shrub *Empetrum hermaphroditum*, disease levels are patchily distributed between hummocks (low disease) and intervening depressions (high disease) that create variations in the persistence of snow cover. *E*. *hermaphroditum* is known to have an allelopathic effect on other species [35] and, consequently, any change in its density is likely to trigger a cascade of changes in the whole ecosystem [36].

## Research challenges and priorities

Just as some have advocated for long-term studies to understand the impact of climate change on plant communities [37], here, we stress the importance and value of large-scale, long-term studies of natural host–pathogen metapopulations. Certainly, many ecological and evolutionary questions can be at least partly addressed through “snap-shots” of multiple populations taken at a single point in time. However, studies extending over decades provide an essential resource for separating the impact of short-term random year-to-year climatic fluctuations from the longer-term trends that will drive ecological and evolutionary change into the future. The size [hundreds to thousands of populations; [28]] and duration [decades] needed for such studies are very significant in making their funding increasingly difficult. To achieve long-term viability, such studies need to embrace a major teaching role and encourage shorter-term inputs from students, postdoctoral researchers, and specialists against an epidemiological/evolutionary core maintained by a nucleus of committed researchers.

Climatic elements are intertwined, generating a multifactorial phenomenon of climate change. While changes in “headline” parameters—mean and maximum temperature, and precipitation attract attention, for pathogens, plants and their interaction, subtler changes, or interactions among the elements may be equally or even more important, primarily attributable to trade-offs in species adaptation. Unfortunately, the identity of all of these factors, for example, day/night temperature changes, shifts in seasonal distributions in precipitation and temperature, and their relevance to particular interactions and broader environments is not always predictable a priori. Furthermore, often, the first signs of change are seen at the microenvironmental level—a level below the threshold of most publicly available climate-related data sets. The very rapid development of a broad range of miniaturized environmental sensors, together with effective reporting networks, provides a cost-effective way forward. However, knowing what the right questions are to ask and being able to formulate these in a way that basic and applied researchers and highly skilled data analysts can understand and test experimentally is an inevitable challenge facing our increasingly multidisciplinary science efforts.

Lack of an adequate understanding of the adaptive patterns and mechanisms of plants, pathogens, and their interactions to climate change is a major barrier to predicting the likely response of both agricultural and wild plant communities. Yet, currently, there is increasing interest in countering the threat posed by disease in agriculture through application of eco-evolutionary approaches that mirror a widely recognized explanation for generally lower disease impacts in wild pathosystems [38,39]. This approach is attractive because it provides a vision of reduced epidemic frequency and amplitude, greater yield stability, lowered pathogen evolutionary change, reduced pesticide use, and overall increased sustainability. Will this still be the case as agricultural pathogens respond to environmental change?

Species adaptation to fluctuating or directional climate changes is likely to be driven by a combination of quasi-genetic and genetic mechanisms leading to selection for plasticity or novel mutations [40]. This occurs against potential shifts in the niche breadth of pathogens and their host plants [5]. There is then an urgent need to empirically study the adaptive responses of pathogens and their hosts to climate changes using cutting-edge technology applied to a combination of approaches such as experimental evolution in the laboratory or field [10,41]. Such studies built around long-term, real-world pathogen–host epidemiological and evolutionary studies will help the development of new models that account for the future climatic preferences and niche breadths of plants, pathogens, and their interactions. Given that pathogen thermal tolerances can evolve and that many host resistance genes are also thermally sensitive, a significant challenge into the future will be to account for the consequences of changing thermal tolerances especially where mismatches that lead to either increasing resistance or aggressiveness develop. In wild pathosystems, any change in climatic conditions can impact host, pathogen, and their interactions and therefore may have marked community effects. In contrast, in agricultural systems, some changes such as incremental variation in precipitation may have little effect as changes in irrigation practices may ameliorate them without much difficulty. Only those changes favoring pathogens, and for which there are no current intervention practices, will need greater human attention.

## Conclusions

The impact of climate change on disease incidence and severity and on the geographic distribution of plant pathogens is hugely important for both agricultural production and the diversity and structure of natural communities. Some aspects of the impacts—particularly with respect to the distribution and severity of agricultural pathogens—appear to be relatively simple to predict and mitigate. However, in the case of natural plant–pathogen interactions where community dynamics are complex and uncontrolled by humans, the outcome of the cascade of interactions that may be precipitated by climate change remains far less clear and unexplored. Understanding these patterns will play a vital role in developing an effective response for biodiversity conservation of natural communities, especially those that currently sit astride major environmental boundaries or in areas, such as the boreal zone, where climate change is expected to have a particularly pronounced impact.
